# Development and validation of age- and sex-specific reference intervals for serum vitamin B5 in Henan pediatric population by LC-MS/MS

**DOI:** 10.3389/fnut.2025.1698679

**Published:** 2025-12-11

**Authors:** Yusheng Luan, Tiewei Li, Fatao Lin, Xiaojuan Li, Junmei Yang

**Affiliations:** 1 Children's Hospital Affiliated to Zhengzhou University, Zhengzhou, Henan, China; 2 Zhengzhou Key Laboratory of Children's Infection and Immunity, Department of Clinical Laboratory, Children's Hospital Affiliated to Zhengzhou University, Zhengzhou, Henan, China; 3 Key Laboratory of Children's Infection and Immunity, Department of Neonatal Medicine, Children's Hospital Affiliated to Zhengzhou University, Zhengzhou, Henan, China

**Keywords:** vitamin B5, reference intervals, validation, children, Henan

## Abstract

**Background and objective:**

Reference intervals (RIs) for serum vitamin B5 (pantothenic acid, B5) specific to the Henan pediatric population have not been established. Current clinical practice, in China continues to rely on reference thresholds derived from international populations. However, these generalized intervals may lack diagnostic accuracy and relevance when applied to domestic children. This study aims to establish age- and sex-specific RIs for serum B5 in Henan children aged 1–17 years, thereby enhancing the reliability of clinical assessments and nutritional evaluations.

**Methods:**

This cross-sectional study involved 1,998 healthy children who underwent routine physical examinations between January 2022 and March 2025. Serum B5 concentrations were quantified using liquid chromatography–tandem mass spectrometry (LC–MS/MS) system. Data exhibiting non-normal distribution are expressed as median and interquartile range (IQR) [M (P_25_, P_75_)]. Age- and sex-related differences were analyzed using the Mann–Whitney *U* test or Kruskal–Wallis test, as appropriate. RIs validation was performed using a subsequent cohort (*n* = 547) examined from March to May 2025.

**Results:**

Age and sex were identified as significant factors influencing serum B5 concentrations in the pediatric cohort. The study population had a predominance proportion of male participants (59.01%, 1,179/1,998), and most subjects were under the age of 12, reflecting a primarily prepubescent sample (93.59%, 1,870/1,970). Based on these demographic characteristics, further stratified analyses were conducted to establish age- and sex-specific RIs for serum B5 levels. For male children, the RIs were 31.57–169.31 ng/mL for the 1–5-year age group, 24.23–127.57 ng/mL for those aged 6–11 years, and 25.36–95.37 ng/mL for those aged 12 and older. Female participants showed RIs of 31.10–166.20 ng/mL, 23.93–114.79 ng/mL, and 18.56–122.55 ng/mL for the same age categories. These stratified intervals highlight the importance of considering both age and sex in the clinical interpretation of pediatric B5 measurements. Validation results confirmed the reliability of the established reference intervals, demonstrating a minimum conformity rate of 93.94%, which meets the 90% threshold stipulated by Chinese industry standards.

**Conclusion:**

This study provides the first comprehensive set of age- and sex-specific RIs for serum B5 in Chinese children aged 1–17 years and has validated them in a representative cohort.

## 1 Introduction

Vitamin B5 functions as an essential precursor in the biosynthesis of coenzyme A (CoA), a pivotal molecule in pediatric energy metabolism, fatty acid synthesis, and neurotransmitter production (1, 2). Although routine dietary intake typically satisfies daily B5 requirements, establishing population-specific RIs for serum B5 is critical for identifying deficiency states linked to growth retardation (3), dermatitis (4), and neurodegenerative diseases (5). This is particularly important for reducing the risk of misdiagnosis arising from symptoms common on deficiencies in other vitamins. Additionally, excessive supplementation has been shown to lead to gastrointestinal complications such as diarrhea, and abdominal cramps (6). These dual clinical implications underscore the necessity for validated pediatric RIs to improve diagnostic accuracy and therapeutic interventions.

Given the well-established physiological role of B5 in children's growth regulation, precise quantification is clinically essential. LC-MS/MS is recognized as the optimal method for assessing B5 in children. This technique offers significant clinical advantages, including minimal sample volume requirements-especially crucial for neonates and infants with limited tolerance for blood sampling and exceptional analytical precision (7–9). However, substantial discrepancies remain between internationally accepted RIs and the biochemical profiles of children in Henan Province, highlighting the urgent need for province-specific RIs to enhance diagnostic accuracy and prevent nutritional mismanagement in this region.

The development of pediatric B5 RIs requires careful consideration of developmental physiological variations, including age-dependent shifts in metabolic demands and sex-specific differences in nutrient utilization. It is essential to control for key factors, such as dietary intake, renal clearance efficiency, and pubertal hormonal regulation, to minimize their confounding effects and ensure accurate interpretation of biological variability.

## 2 Methods

### 2.1 Study design and participants

This cross-sectional study was based on retrospectively collected clinical data from pediatric patients who underwent routine health evaluations at Henan Children's Hospital (affiliated with Zhengzhou University) between January 2022 and March 2025.

This study was conducted in accordance with the Chinese health industry standard WS/T 402−2024 (National Health Commission of the People's Republic of China) for defining, establishing, and verifying RIs for quantitative laboratory assays (hereinafter referred to as the Chinese health industry standard) (10). Accordingly, the study design and participant selection criteria were derived from its guidelines. The study cohort consisted of 1,998 children aged 1–17 years including 1,179 males and 819 females. According to the standard's recommendations, participants were included if they were aged 1–17 years and documented in health examination records. Exclusion criteria, which were defined based on the standard, comprised: (1) malnutrition, (2) short stature, (3) hepatic or renal disorders, (4) vitamin B5 supplementation within 30 days prior to blood collection, and (5) absence of documented vitamin test results.

Outliers were removed using the D/R ratio method as recommended, where D represents the absolute difference between an extreme observation (either the largest or smallest) and the next largest (or smallest) observation, and R represents the range of all observations including the extreme value. A cutoff value of 1/3 was applied: if the difference D is equal to or greater than one-third of the range R, the extreme observation is deleted (10).

For validation of the established RIs, an independent cohort of 547 children (329 males, 218 females) who underwent health examinations between 19 March and 28 May 2025 was subsequently enrolled, representing 27.4% of the original cohort. The validation process adhered to the Chinese health industry standard for verifying RIs. The Ethics Review Board of Henan Children's Hospital waived informed consent requirements due to the retrospective nature of this study.

### 2.2 Cohort partitioning strategy

The study cohort was stratified into three age groups (1–5 years, 6–11 years, and 12–17 years), encompassing both sexes. Concurrently, based on the seasonal classification of the North China Meteorological Zone, the study period was partitioned into four climatic intervals: spring (March-May), summer (June–August), autumn (September–November), and winter (December–February).

### 2.3 Clinical data collection

Demographic and biochemical parameters, including age, sex, and serum B5 concentrations, were retrospectively extracted from the institutional electronic medical records (EMRs). Serum B5 levels were quantified using the Waters ACQUITY UPLC I-Class Plus system coupled with a Xevo TQ-S Micro tandem mass spectrometer (Waters Corporation). Serum samples were processed following the manufacturer's instructions (Hanahao Biotech, Tianjin, China), involving solid-phase extraction and chromatographic separation, with a 5 μL injection volume.

Mass spectrometric detection was performed in positive electrospray ionization (ESI+) mode, applying optimized instrumental settings: capillary voltage of 3.0 kV, ion source temperature of 150 °C, desolvation temperature of 500 °C, desolvation gas flow rate of 1,000 L/h, and cone gas flow of 50 L/h. Quantitative analysis was carried out using multiple reaction monitoring (MRM) transitions specific to the target analyte, with calibration against certified reference materials. Data acquisition and analysis were performed using MassLynx version 4.2 software.

The limit of quantification (LOQ) for vitamin B5 was 3 ng/mL. At this concentration, the maximum deviation was −14.1% and CV = 7.09%, both within the acceptance criteria (±15% deviation, CV ≤ 20%). Intra- and inter-day precision (9, 60, and 450 ng/mL; *n* = 3 days) yielded CVs ≤ 10.60% and ≤ 8.10%, respectively. Recovery rates ranged from 85.05% to 114.79%, meeting the acceptable range (85–115%).

### 2.4 Statistical analysis

Statistical analyses were performed using SPSS version 27.0. Non-normally distributed variables are presented as medians with interquartile ranges (P_25_, P_75_). Descriptive statistics summarized serum B5 concentrations across different age groups, sexes, and sample collection seasons. The Mann-Whitney U test was applied for comparisons between two independent groups, while the Kruskal-Wallis test was used for comparisons among three or more groups. Multivariate linear regression analysis was conducted to evaluate the independent effects of sex, age group, and season on serum vitamin B5 concentrations, with standardized regression coefficients (β) and corresponding *P*-values reported. Graphical representations were generated using GraphPad Prism 8 (GraphPad Software Inc.). A two-sided *P*-value < 0.05 was considered statistically significant.

## 3 Results

### 3.1 Characteristics of participants

**Figure 1:**
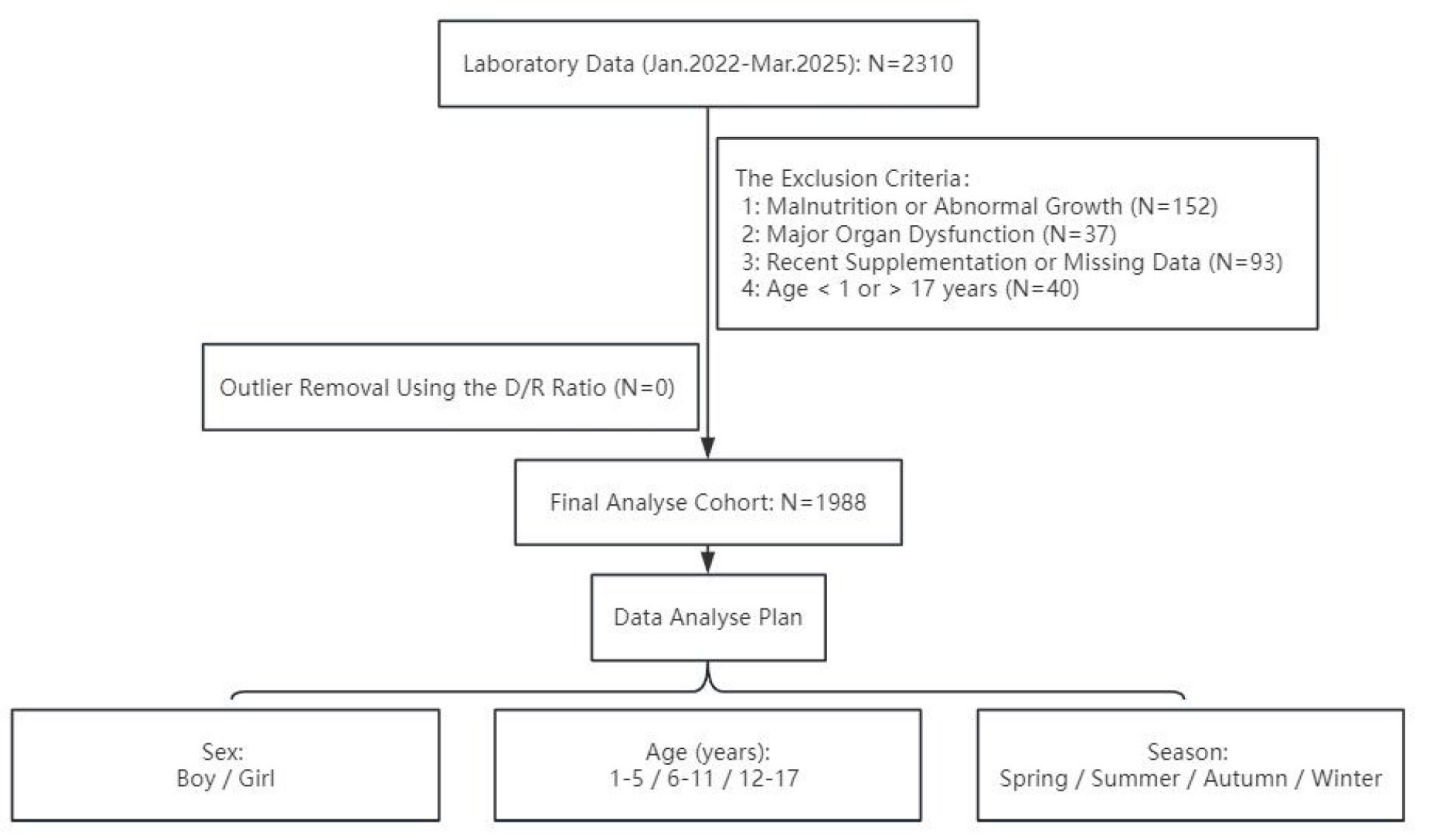
Flowchart of participant screening, outlier assessment, and analytical cohort formation.

A total of 2,310 children who underwent health examinations at Henan Children's Hospital between January 2022 and March 2025 were initially enrolled. According to the exclusion criteria outlined in Section 2.1, 322 children were excluded during preliminary screening. Further refinement based on the methodology prescribed by the Chinese health industry standard resulted in a final cohort of 1,988 children included in the study, as illustrated in Figure 1.

As presented in Table 1, the majority of participants (47.95%, 958/1,998) were under 6 years of age. A consistent male predominance was observed across all age groups, with the most pronounced sex difference seen in the 12–17 years group (male-to-female ratio: 2.12:1). Statistically significant sex-based differences in serum B5 concentrations were identified, with boys consistently exhibiting higher median levels than girls across all developmental stages (Δ Median: 2.48–4.65 ng/mL; all *P* < 0.05). Meanwhile, both sexes exhibited a gradual decline in median serum B5 levels (Median [P_25_-P_75_]) with age, which aligns with known physiological changes in pantothenate metabolism during maturation.

**Table 1:** Characteristics of subjects.

**Stratification, age (years)**	**Subgroup, gender**	***n* (%)**	**B5 median (P_25_-P_75_) (ng/mL)**	Gender effect (boy-girl Δ^*^) (ng/mL)	***P***
1–5		958 (47.95%)			
	Boy	545 (56.89%)	62.01 (48.45–84.80)	+4.65	0.016
	Girl	413 (43.11%)	57.36 (45.99–79.77)		
6–11		912 (45.65%)			
	Boy	547 (59.98%)	47.22 (38.27–59.70)	+2.48	0.036
	Girl	365 (40.02%)	44.74 (36.43–56.79)		
12–17		128 (6.40%)			
	Boy	87 (67.97%)	41.12 (34.33–52.43)	+4.16	0.013
	Girl	41 (32.03%)	36.96 (27.12–47.14)		
Total		1,998 (100%)	51.73 (41.14–70.48)		

B5, vitamin B5.

^*^The median B5 level in boys minus the median B5 level in girls.

### 3.2 Gender variation in B5 levels

**Figure 2:**
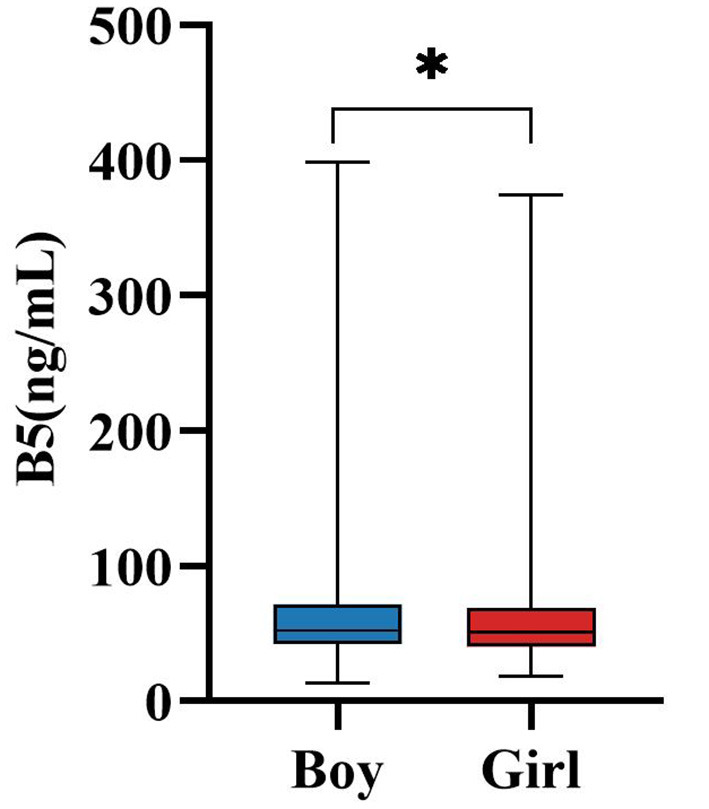
Gender variation in B5 levels. *P < 0.05; ns, no significant; B5, vitamin B5.

Sex-related differences in serum vitamin B5 concentrations were observed in our cohort. Table 2 summarizes sex-specific distributions of serum B5 concentrations among the 1,998 children, including 1,179 boys and 819 girls. Median B5 levels were slightly higher in boys (52.44 ng/mL, IQR: 42.13–71.73) compared to girls (50.79 ng/mL, IQR: 40.32–69.10). Statistically significant differences between sexes were evident both in the overall cohort and across all age-stratified subgroups (*P* < 0.05 for each comparison), with boys consistently demonstrating higher median values. These sex-based variations are further illustrated in Figure 2, where comparative analysis across developmental stages reinforced the presence of significant inter-sex differences in serum B5 concentrations (*P* < 0.05). Consistent with these descriptive findings, multivariate linear regression analysis (Supplementary Table 1) further confirmed that sex was an independent predictor of serum B5 concentrations (standardized β = −0.047, *P* = 0.028) after adjusting for age group and season, although the effect size was relatively small.

**Table 2:** Gender variation in B5 levels.

**Variables**	**Boy (*****n*** = **1,179)**			**Girl (*****n*** = **819)**			**Z**	***P***
	**P**_**25**_	**P**_**50**_	**P**_**75**_	**P**_**25**_	**P**_**50**_	**P**_**75**_		
B5 (ng/mL)	42.13	52.44	71.73	40.32	50.79	69.10	−2.35	0.019

B5, vitamin B5.

### 3.3 Age variation in B5 levels

**Figure 3:**
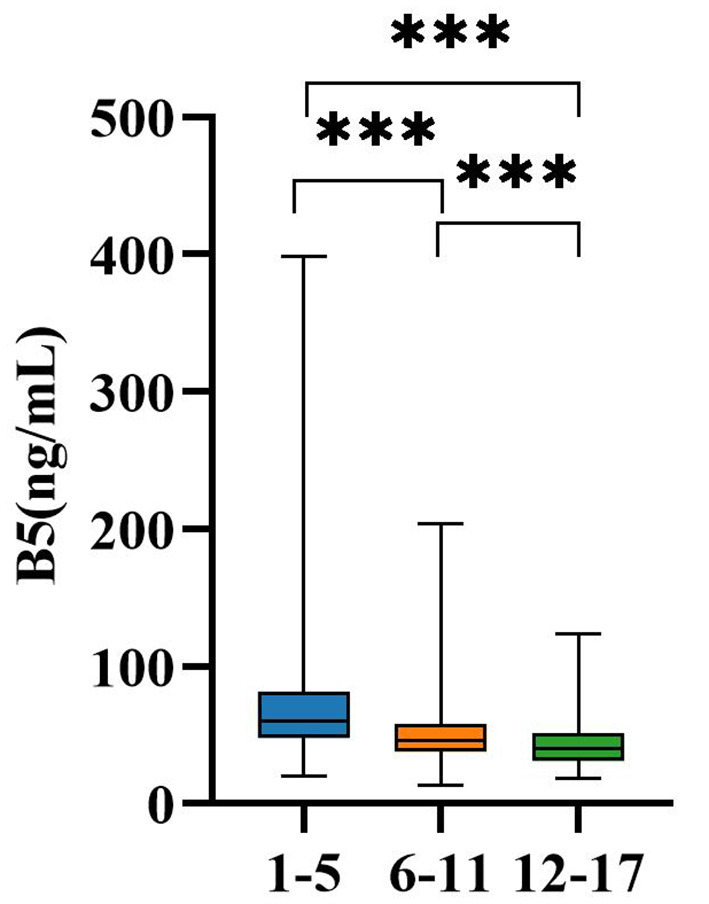
Age variation in B5 levels. ***P < 0.001; ns, no significant; B5, vitamin B5.

Table 3 categorizes participants into three distinct age groups: 1–5 years, 6–11 years, and ≥12 years. The largest proportion of the study population was in the 1–5-year group (*n* = 958), while the ≥12-year category had the fewest participants (*n* = 128). Serum B5 concentrations demonstrated a clear inverse relationship with age, with median (IQR) values of 60.22 ng/mL (47.53–81.95) in the 1–5-year group, 45.95 ng/mL (37.55–58.71) in the 6–11-year group, and 39.58 ng/mL (31.16–51.24) in those aged ≥12 years. This decline trend is visually represented in Figure 3, highlighting a significant reduction in B5 levels with advancing age (*P* < 0.001 for all pairwise comparisons). To further substantiate these observations, multivariate linear regression analysis (Supplementary Table 1) was conducted, adjusting for sex and season. The results identified age group as a strong and independent predictor of serum B5 concentrations (standardized β = −0.302, *P* < 0.001), confirming that the association between age and B5 levels persisted after accounting for potential confounders. Collectively, these findings suggest that the age-related decline in B5 concentrations reflects intrinsic maturational adaptations in pantothenate metabolism—such as reduced coenzyme A turnover and evolving energetic requirements during growth and development.

**Table 3:** Age variation in B5 levels.

**Variables**	**1–5 years (*****n*** = **958)**			**6–11 years (*****n*** = **912)**			**12–17 years (*****n*** = **128)**			**Z**	***P***
	**P**_25_	**P**_50_	**P**_75_	**P**_25_	**P**_50_	**P**_75_	**P**_25_	**P**_50_	**P**_75_		
B5 (ng/mL)	47.53	60.22	81.95	37.55	45.95	58.17	31.16	39.58	51.24	283.26	< 0.001

B5, vitamin B5.

### 3.4 Seasonal variation in B5 levels

**Figure 4:**
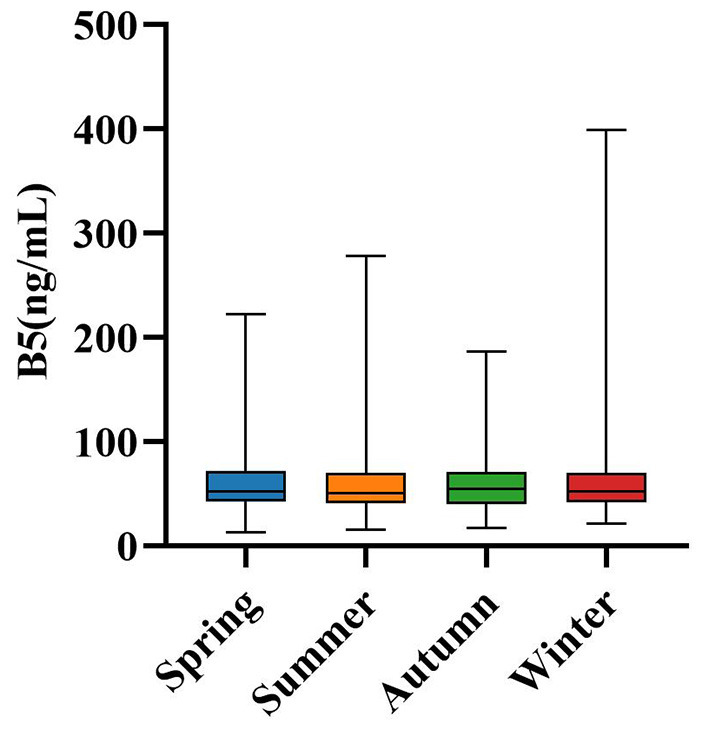
Season variation in B5 levels. All comparisons were not statistically significant. B5, vitamin B5.

Table 4 presents the distribution of serum B5 concentrations across different seasons. Among pediatric patients who underwent routine health evaluations at our institution between January 2022 and March 2025, the majority were assessed during the summer (*n* = 747) and winter (*n* = 509), while the autumn season accounted for the smallest subgroup (*n* = 247). Despite this seasonal imbalance in participant numbers, median serum B5 levels remained consistent across all four seasons: spring [52.38 ng/mL [42.22, 71.80]], summer [50.23 ng/mL [40.75, 69.81]], autumn [54.42 ng/mL [39.91, 70.80]], and winter [52.12 ng/mL [41.30, 70.41]]. The Kruskal–Wallis test revealed no statistically significant differences among seasons (*P* = 0.487), suggesting that serum B5 concentrations do not vary seasonally in this population. Consistently, multivariate linear regression analysis (Supplementary Table 1) showed that after adjusting for age group and sex, season exerted no significant independent effect on serum B5 concentrations, as indicated by an almost null standardized β (−0.001) and a non-significant *P*-value (0.972). This conclusion is further supported by Figure 4, which illustrates overlapping interquartile distributions across all seasonal categories. The apparent lack of temporal fluctuation suggests that pediatric B5 regulation is predominantly governed by intrinsic physiological mechanisms rather than external environmental factors, in line with its essential role in maintaining table CoA-dependent metabolic activity.

**Table 4:** Season variation in B5 levels.

**Variables**	**Percentiles**	**Spring**	**Summer**	**Autumn**	**Winter**	**Z**	***P***
		**(*****n*** = **495)**	**(*****n*** = **747)**	**(*****n*** = **247)**	**(*****n*** = **509)**		
B5 (ng/mL)	P_25_	42.22	40.75	39.91	41.30	3	0.487
	P_50_	52.38	50.23	54.42	52.12		
	P_75_	71.80	69.81	70.80	70.41		

B5, vitamin B5.

### 3.5 Reference intervals of B5 according to age and gender group

Table 5 outlines the age- and sex-stratified RIs for serum B5 in children aged 1–17 years. In the youngest cohort (1–5 years), boys exhibited an RI of 31.57–169.31 ng/mL, while girls showed a comparable range of 31.10–166.20 ng/mL. In the 6–11-year group, boy RIs narrowed to 24.23–127.57 ng/mL, with females demonstrating a similar contraction to 23.93–114.79 ng/mL. Notably, in the 12–17-year cohort distinct sex-specific difference emerged: boys exhibited a higher lower limit (25.36 ng/mL) but tighter upper boundary (95.37 ng/mL), whereas females displayed broader range (18.56–122.55 ng/mL). These progressive age-dependent adjustments in RIs reflect shifts in metabolic priorities during development, with more pronounced sex differences emerging during adolescence.

**Table 5:** Recommended reference intervals for different age groups and genders (P_2.5_, P_97.5_).

**Variables**	**1–5 years**		**6–11 years**		**12–17 years**	
	**Boy**	**Girl**	**Boy**	**Girl**	**Boy**	**Girl**
B5 (ng/mL)	31.57–169.31	31.10–166.20	24.23–127.57	23.93–114.79	25.36–95.37	18.56–122.55

B5, vitamin B5.

### 3.6 Validation of reference intervals

To validate the applicability of our established RIs, we selected children who underwent health examinations at our hospital from 19 March to 28 May 2025. These children strictly met the aforementioned exclusion criteria, and outliers were removed using the same methods as previously described, resulting in a final cohort of 547 subjects. The new samples were stratified according to the same age-group classification scheme, with boys distributed across the three age groups as follows: 110 (1–5 years), 186 (6–11 years), and 33 (12–17 years), and girls as follows: 84, 10, and 27. Subsequently, we applied the new sample data to the established RIs and found that the proposed B5 RIs demonstrated high applicability for children aged 1–17 years in Henan Province (Table 6). Although the group with the highest proportion of values outside the RIs was the female group aged 1–5 years, 93.94% of the samples in this group still fell within the established RIs, a rate closely approximating the ideal 95%, indicating that the RIs are generally well-suited for the target population.

**Table 6:** Validation of reference intervals.

**Variables**	**1–5 years (*****n*** = **194)**		**6–11 years (*****n*** = **293)**		**12–17 years (*****n*** = **60)**	
	**Boy**	**Girl**	**Boy**	**Girl**	**Boy**	**Girl**
*n*	110	84	186	107	33	27
In range^*^ (%)	108 (98.18%)	79 (94.05%)	178 (95.70%)	104 (97.20%)	31 (93.94%)	27 (100%)

^*^In range denotes the proportion of new samples falling within the established reference intervals.

## 4 Discussion

B5, a water-soluble micronutrient essential for energy transduction, circulates primarily as free pantothenate and 4′-phosphopantetheine, which is a precursor for CoA biosynthesis (11, 12). Over 5% of human metabolic enzymes, particularly those involved in fatty acid oxidation and acetylcholine synthesis, rely on CoA-derived moieties for catalytic activity (13, 14). As humans lack the ability to synthesize B5 endogenously, dietary intake serve as the primary source, with minimal contributions from gut microbiota metabolism (15, 16).

This study revealed a significant sex-based differences in B5 levels. Given that B5 intake primarily depends on diet and is minimally influenced by gut microbiota, the observed variation may be attributed to dietary preferences. Boys typically consume more animal-derived foods than girls, which can lead to higher B5 concentrations. Additionally, this disparity may be linked to androgen-mediated regulation of pantothenate kinase activity, the rate-limiting enzyme in CoA biosynthesis. Animal studies have shown that testosterone enhances the expression of pantothenate kinase 1 in liver tissue, which may explain the elevated B5 levels in adolescent males (17, 18). In contrast, estrogen may inhibit renal reabsorption of pantothenic acid, potentially contributing to lower B5 levels in adolescent females (19). These findings align with previous research, further supporting the role of biological sex in determining B5 concentrations (20, 21).

The observed inverse relationship between age and B5 concentrations reflects developmental metabolic shifts. During early childhood, intestinal activity is high and nutrient absorption is efficient, with intake generally exceeding consumption, leading to relatively elevated serum B5 levels. As children age, the activity of the tricarboxylic acid (TCA) cycle stabilizes, the renewal rate of CoA decrease, and the metabolic turnover of B5 increases, resulting in lower serum levels. Furthermore, hormonal changes during puberty and increased tissue demand for CoA may further lower serum B5 concentrations. With the maturation of tissue distribution and excretion mechanisms, the body's regulation of B5 becomes more refined and controlled, which may also contribute indirectly to the decline in serum B5 levels.

Our analysis identified no significant seasonal influence on B5 levels which can be attributed to two primary factors: First, the wheat-based dietary pattern in Henan Province provides a consistent intake of pantothenic acid for children (11, 22). Second, advancements in agricultural technology and global food distribution systems ensure year-round availability of diverse food sources, minimizing seasonal fluctuations in serum B5 concentrations. Consequently, no seasonal effect on B5 levels was observed.

In addition, international data provide a valuable context for interpreting our findings. According to Andraos et al. (Nutrients), plasma pantothenic acid concentrations in Australian children measured by UHPLC–MS/MS were 182.53 (153.72–219.27) nM in boys [≈39.98 [33.69–48.06] ng/mL] and 165.17 (138.68–196.33) nM in girls [≈36.22 [30.40–43.03] ng/mL], showing slightly higher values in males (23). These results are comparable to our data in Henan children, where boys consistently exhibited higher median B5 levels than girls across age groups. Similarly, the reference interval reported by the Children's Mercy Hospital (U.S.)−12–34 ng/mL for children aged 1–17 years—falls within a comparable range, further supporting the reliability of our results despite methodological differences (plasma vs. serum, LC–MS/MS vs. UHPLC–MS/MS). This alignment underscores the external validity and broader applicability of our established reference intervals.

To further substantiate the robustness of our findings, the established RIs were validated using an independent cohort of 547 children from the same region. The validation results aligned well with expectations, demonstrating high applicability across age and sex subgroups. Notably, the lowest conformity rate observed across all subgroups was 93.94%, exceeding the 90% threshold set by the Chinese health industry standard, which supports the applicability of these RIs for assessing serum B5 levels in children of corresponding ages.

Building on the impact of age and sex, this study established RIs for B5 in children from Henan Province. These RIs enhance diagnostic accuracy for identifying B5 deficiency or excess, thereby reducing the risk of misdiagnoses such as developmental delays and dermatitis, which are associated with B5 disorders.

However, this study has certain limitations. The RIs were established based on children from Henan Province, which may limit generalizability to other regions. As a single-center retrospective study, detailed dietary data, supplement intake, genetic information (including pantothenate transporter polymorphisms), endocrine markers, and other B5-related biochemical measurements were not available, restricting the assessment of factors contributing to interindividual variability. Longitudinal follow-up was also not conducted, limiting the ability to observe intra-individual changes over time.

Despite these limitations, our study provides real-world children B5 RIs for Henan Province, serving as a foundation for future research in other regions and populations. We are also collecting data from pediatric patients with suspected B5 deficiency to explore clinical cutoff values. Future multi-center, prospective studies incorporating dietary, endocrine, and additional biochemical assessments will help refine and validate these reference intervals, enhancing their clinical applicability.

## 5 Conclusion

Age-stratified variations in B5 status were systematically assessed through LC-MS/MS quantification, establishing region-specific RIs for pediatric populations. The cohort was divided into three developmental phases: 1–5 years, 6–11 years and ≥12 years, with corresponding RIs for boys (31.57–169.31, 24.23–127.57, 25.36–95.37 ng/mL) and girls (31.10–166.20, 23.93–114.79, 18.56–122.55 ng/mL). These RIs were subsequently validated, demonstrating coverage that met or even exceeded our expectations. These age specific thresholds offer valuable references for addressing regional nutritional requirements and support evidence-based clinical decision-making in the prevention of pediatric metabolic disorders.

## Data Availability

The original contributions presented in the study are included in the article/Supplementary material, further inquiries can be directed to the corresponding authors.
