# Supplementary material for: Development and validation of age- and sex-specific reference intervals for serum vitamin B5 in Henan pediatric population by LC-MS/MS
Source: Front Nutr. 2025 Dec 11;12:1698679. doi: 10.3389/fnut.2025.1698679 (PMC12739956; doi:10.3389/fnut.2025.1698679)
Supplement: Supplementary file 1 [file Table_1.docx]

Supplementary Table 1. Multivariate linear regression analysis of serum B5 concentrations in children

| Variables | Standardized β | *P* |
| --- | --- | --- |
| Sex | -0.047 | 0.028 |
| Age | -0.302 | ＜0.001 |
| Season | 0.001 | 0.972 |

Notes: Age group was coded as 1 = 1-5 years, 2 = 6-11 years, and 3 = 12-17 years.

Sex was categorized as male or female.

Season was divided into four groups corresponding to the four seasons.

Standardized β allows comparison of the relative effect sizes of each variable.
